# Implementing mental health support teams in schools and colleges: the perspectives of programme implementers and service providers

**DOI:** 10.1080/09638237.2023.2278101

**Published:** 2023-11-08

**Authors:** Jo Ellins, Lucy Hocking, Mustafa Al-Haboubi, Jennifer Newbould, Sarah-Jane Fenton, Kelly Daniel, Stephanie Stockwell, Brandi Leach, Manbinder Sidhu, Jennifer Bousfield, Gemma McKenna, Catherine Saunders, Stephen O’Neill, Nicholas Mays

**Affiliations:** a Health Services Management Centre, School of Social Policy, University of Birmingham, Birmingham, England; b RAND Europe, Cambridge, England; c Department of Health Services Research and Policy, London School of Hygiene and Tropical Medicine, London, England; d Institute for Mental Health, University of Birmingham, Birmingham, England; e Department of Public Health and Primary Care, University of Cambridge, Cambridge, England

**Keywords:** mental health, children, early intervention, education, implementation, CBT, paraprofessionals

## Abstract

**Background:**

Between 2018 and 2025, a national implementation programme is funding more than 500 new mental health support teams (MHSTs) in England, to work in education settings to deliver evidence-based interventions to children with mild to moderate mental health problems and support emotional wellbeing for all pupils. A new role, education mental health practitioner (EMHP), has been created for the programme.

**Aims:**

A national evaluation explored the development, implementation and early progress of 58 MHSTs in the programme’s first 25 ‘Trailblazer’ sites. This paper reports the views and experiences of people involved in MHST design, implementation and service delivery at a local, regional and national level.

**Methods:**

Data are reported from in-depth interviews with staff in five Trailblazer sites (n = 71), and the programme’s regional (n = 52) and national leads (n = 21).

**Results:**

Interviewees universally welcomed the creation of MHSTs, but there was a lack of clarity about their purpose, concerns that the standardised CBT interventions being offered were not working well for some children, and challenges retaining EMHPs.

**Conclusions:**

This study raises questions about MHSTs’ service scope, what role they should play in addressing remaining gaps in mental health provision, and how EMHPs can develop the skills to work effectively with diverse groups.

## Introduction

Many mental health problems are first experienced during childhood or adolescence (Kessler et al., 2007) and the proportion of children reporting poor mental health has increased significantly in recent years. In England, a national survey published in 2020 estimated that one in six children had a probable mental health problem, up from one in nine in 2017 (NHS Digital, 2020). Covid-19 and restrictions to control the spread of the virus simultaneously increased factors associated with psychological distress (e.g. loneliness, family conflict, bereavement) and reduced access to activities that promote mental wellbeing and wider sources of support (Bunn & Lewis, 2021; El-Osta et al., 2021). These detrimental effects have particularly affected children already at increased risk of experiencing mental health problems (Mansfield et al., 2021; Newlove-Delgado et al., 2021).

Mental health difficulties in childhood can have a profound and enduring impact on quality of life, relationships, academic success and employment opportunities (Copeland et al., 2015; Goodman et al., 2011; Lereya et al., 2019). Despite this, children and families have long reported facing difficulties accessing support, with mental health services often experienced as patchy, fragmented and over-stretched, and lengthy waits before they are assessed and/or care is provided. (Care Quality Commission, 2017; Crenna-Jennings, 2021). As a result, many children’s mental health problems significantly worsen before they receive appropriate help (Young Minds, 2018).

Most children spend more time in school than any other place outside their home, and parents concerned about their child’s mental health turn to teachers for help and advice more often than any other professional group (Newlove-Delgado et al., 2015). There has been growing recognition of the important role that schools can and do play in promoting emotional wellbeing, and in identifying and supporting children with mental health problems. Developing ‘whole school approaches’ to mental health and wellbeing has emerged as a shared goal for mental health and education policy in England (Department for Education, 2021; Department of Health & NHS England, 2015). Many schools offer mental health support on-site, most commonly counselling or educational psychologist support, but provision is highly variable (Marshall et al., 2017). While children differ in their help-seeking and preferences for mental health support, more would like to access help within their school than are currently able to do so (Children’s Commissioner, 2021).

### Mental health support teams

The Children and Young People’s Mental Health Implementation programme was launched in 2018 by the Department of Health and Department for Education, to take forward the proposals set out in the *Transforming Children and Young People’s Mental Health Provision* Green Paper (Department of Health & Department for Education, 2017). With the aim of improving prevention and early intervention for children’s mental health within schools and further education colleges in England, it comprised the following elements:

- Funding the creation of new mental health support teams (MHSTs) to work in schools and colleges, with three core functions: providing evidence-based psychological interventions for mild to moderate mental health problems; working with school and college staff to promote emotional wellbeing across the setting; and supporting and advising staff and parents to help children (e.g. with more complex mental health needs) access other services and sources of support.
- Encouraging schools and colleges to appoint a senior staff member to lead and coordinate the (further) development of a ‘whole school’ approach to mental health, with grants available for leads to attend training to support them in the role.
- Piloting a four-week waiting time for access to specialist NHS children and young people’s mental health services in selected sites.

A systematic review of psychosocial interventions (including cognitive behaviour therapy (CBT), parent training and family-based interventions) for children and adolescents with common mental health difficulties was undertaken to inform programme design, which reported modest, persistent improvements compared to active controls (Pilling et al., 2020). Interventions delivered by paraprofessionals (defined as “school professionals or non-mental health professionals with intervention-specific training” (Pilling et al., 2020, p.5)) were as or more effective for anxiety and depressive disorders, with evidence suggesting that effectiveness was greatest where paraprofessionals were trained in specific interventions, focused on less severe disorders, and supported by continuing supervision.

While MHSTs have flexibility in their staffing composition, it is expected that around half of the team will be comprised of education mental health practitioners. This is a new paraprofessional role created for the programme. EMHPs undergo a 12-month university training programme combining classroom learning and supervised placements in education settings. This includes training in the delivery of brief, low-intensity interventions, based on CBT principles, for mild-moderate anxiety, low mood and behavioural difficulties (Health Education England, 2020). EMHPs are supervised by, and work alongside, more experienced therapists in MHSTs.

Roll-out of the programme is phased, starting with the creation of 58 MHSTs in 25 ‘Trailblazer’ sites, followed by ten more waves between 2019 and 2025. By 2023, more than 400 teams will be operational, serving an estimated three million 5-18 year olds (NHS England, 2022). The programme is being evaluated in two stages. The first stage, the study reported herein, was a process evaluation focusing on the implementation of MHSTs in the programme’s first 25 Trailblazer sites, conducted between October 2019 and May 2022. This was followed by, and informed, a large-scale evaluation of the programme’s outcomes and impact that started in summer 2023.

## Methods

This paper presents qualitative in-depth interview data from the early mixed-method process evaluation that explored the development, implementation and early progress of the programme in the Trailblazer sites. The evaluation was undertaken by a multi-disciplinary team combining expertise in youth mental health, policy evaluation, health services research, qualitative methods, research co-production, routine data analysis and health economics. Interviewees were involved in programme design and/or delivery at a local, regional or national level, and interviews explored their views and experiences of MHSTs, including local delivery models and how these were working in practice, with a focus on drawing out practical learning to support ongoing programme implementation.

### Interviews

Five Trailblazer sites were purposively selected for an in-depth investigation to ensure variation in terms of geographical location, type of organisation leading the MHST service, population characteristics and MHST staffing composition. Project leads in each site was asked to compile a list of individuals involved in the design, management and/or delivery of the MHST service in their area, to include a range of the following: MHST staff; mental health leads in schools and colleges; mental health service providers and commissioners; and staff working in local authority children’s services, voluntary sector organisations and public health teams. Potential participants were approached by a member of the research team, and interviews were undertaken between July 2021 and February 2022. Further interviewees were identified as the research progressed; in later recruitment, the team prioritised groups that were under-represented in the initial interviews to try and achieve a similar range of roles and perspectives across sites. A suite of tailored topic guides was developed (Additional File 1), which explored local governance and management arrangements, service models, staffing, what a ‘typical’ day/week for the MHST involved, training and development needs, relationship building and partnership working, if and how MHSTs were ensuring equity of access for under-served groups, and service sustainability.

Additionally, two rounds of group interviews were carried out with members of the seven regional teams overseeing local implementation of MHSTs in November 2020 – January 2021 and November 2021 – January 2022. In January and February 2022, group interviews were also undertaken with staff from the programme’s national partners (Department of Health and Social Care, Department for Education, NHS England and Health Education England), and other individuals involved in programme delivery at a national level, including course leads from two universities providing EMHP training. Due to Covid-19 restrictions, interviews were carried out remotely via telephone or video-conferencing platform. Participants either completed a consent form in advance, or verbal consent was given and recorded before the interview.

### Data analysis

Interviews were audio recorded, fully transcribed, anonymised and imported into NVivo 12 qualitative data analysis software. Data were analysed thematically using the Framework Method (Ritchie & Spencer, 1994), developed for the purpose of applied policy research. Researchers initially familiarised themselves with the data from the interviews they had conducted. A preliminary coding framework was developed based on initial reading of the interview transcripts, study aims, reviews of relevant literature and discussions at a half-day data analysis workshop. This was then applied to a sample of transcripts, and further codes were developed based on this analysis and subsequent discussions. Five researchers (JN, LH, S-JF, BL and SS) coded the transcripts, meeting fortnightly to discuss the process and ensure consistency in the application of the framework.

A structured template was developed for each researcher to summarise their data, and to support comparative analysis and data synthesis. Two further half-day workshops were held to discuss emerging findings, identify issues requiring further investigation, explore patterns within and between sites and data sets, and incorporate insights from the wider literature.

### Research approvals

The study received ethical approval from the University of Birmingham (ERN_19-1400 - RG_19-190) and London School of Hygiene and Tropical Medicine (Ref: 18040), and from the Health Research Authority (IRAS 270760).

## Results

### Respondents

Interviews were carried out with 71 participants in the five in-depth case study sites, 52 regional leads (12 of whom were interviewed in both rounds of fieldwork, making 40 unique interviewees) and 21 people involved in the design and delivery of the programme nationally. Participant characteristics are shown in Tables 1 and 2. Socio-demographic information was not collected from participants because they were purposively sampled for their knowledge and experience of programme implementation, not for their socio-demographic profile.

**Table 1.** Interviews in in-depth research sites.

Site	Education mental health practitioners	Mental health support team staff (not including EMHPs)	School and college staff	Individuals in governance and/or management roles	Other organisations*	Total number of people interviewed
1	4	3	1	6	0	14
2	5	3	2	2	0	12
3	3	4	2	6	2	17
4	4	3	1	2	2	12
5	3	2	3	3	5	16
Total	19	15	9	19	9	71

* Included voluntary sector organisations, NHS provider trusts and local authorities.

**Table 2.** Interviews with programme regional and national leads.

	Number of people interviewed		
Organisation	First round of regional team interviews	Second round of regional team interviews	National interviews
Department for Education	9	8	6
NHS England and Improvement	13	13	4
Department of Health and Social Care	0	0	4
Health Education England	2	4	2
Training providers	0	0	4
Other	2	1	1
Total	26	26	21

## Findings

We present the findings in relation to four key themes: i) mental health versus wellbeing-oriented service models; ii) MHSTs’ mild to moderate remit; iii) the effectiveness of low-intensity CBT; and iv) staff recruitment and retention.

### Mental health versus wellbeing-oriented service models

The MHST service was nationally specified, but sites had flexibility in how they set up and ran their teams. Broadly, two types of local service models emerged: one more strongly focused on providing therapy to individual children and integration with existing mental health services in the locality; the other giving greater prominence to collaborative work with education settings to develop whole school approaches, with a stronger emphasis on relationship building with educational, voluntary sector and other non-NHS partners:

T*here is a difference I think between a sort of health-led model and a local authority or third sector led model, you can see the differences in terms of, you know, health-based models tend to be very clinical focused, local authority based models tend to try and stretch boundaries around clinical interventions and have a more sort of connection with local authority partners that do whole school approach. (Regional lead)*

Underpinning these models were two different interpretations of the purpose of MHSTs and the wider programme: while some considered it to be further extending children’s mental health services into new settings, others saw the aim as foremost to strengthen the promotion of wellbeing in schools and colleges. In some sites, tensions arose between partner organisations around these competing interpretations:

*We’ve had to work fairly hard at actually saying ‘No, we need to listen to the voice of schools here, this is meant to be a schools facing programme and not just a rolling out of a health programme’. (Site 5, project lead)*

Monitoring data gathered from MHSTs by NHS England (between July and December 2020) and made available to the research team indicated that, on average, MHSTs were spending more of their time delivering therapeutic interventions (52%) than supporting whole-school approaches (24%) or giving advice and liaising with external services (23%). Covid-19 had acted as a barrier to MHSTs’ working with, and physically in, education settings to develop whole school approaches, but our findings suggest this was neither the only, nor potentially the primary, reason why MHSTs were often more focused on one-to-one mental health support. The way in which the programme had been set up was considered by some participants to have given primacy to activities in line with the goals of NHS mental health services rather than those focused on broader notions of wellbeing. This was illustrated by participants, for example, with reference to the programme being funded by NHS England; MHSTs in all but three Trailblazer sites being delivered by an NHS mental health trust (solely or as lead partner in a local collaboration); a perceived dominance in the EMHP national training curriculum on knowledge and skills to deliver individual psychological interventions; and the lack of attention in routine data reporting on capturing whole school activities and their impacts. In this context, educational perspectives and priorities could be overlooked:

*It’s a very NHS dominated space…[We are] trying to bring education to the forefront of every discussion because it does obviously get lost in health. (Regional lead)*

### MHSTs’ mild-moderate remit

Education settings universally welcomed investment in ‘in-house’ mental health support and the focus on early intervention, which came at a time when many were seeing substantial increases in pupil mental health problems as a result of Covid-19. The intention that MHSTs provide support for those with mild-to-moderate mental health problems was not, however, well received by all. Some schools and colleges reported that their staff had the confidence and skills to support children with mild to moderate mental health problems, and so MHSTs were providing ‘more of the same’ rather than increasing the range of mental health needs that could be met. Among these education settings, there was a view that help for children with more complex mental health problems presented a greater need.

A key issue concerned children whose mental health problems neither fitted within MHSTs ‘mild to moderate’ remit, nor met the threshold for accessing specialist services. While concerns about gaps between services pre-dated the pandemic, and indeed were a reason why MHSTs were created, several interviewees commented that a growing number of children were said to be falling into these gaps because of the increase in more serious mental health problems related to Covid-19, and as referral criteria for specialist services tightened to cope with the growing demand for their support. MHST staff were acutely aware of these difficulties and of the lengthy waits for accessing specialist mental health services:

*One is the capacity anyhow in CAMHS [child and adolescent mental health services]. They are really, really struggling, so even if you could prove that a case did belong to them, we know that they won’t get a service straight away. So, there’s a little bit of…tension for us about not wanting to refer a child into a service where we know they’re not going to get a service, basically.* (Site 4, management/governance)

MHSTs were receiving referrals for children with more serious mental health problems, including providing interim support to those on waiting lists for specialist mental health services. They were responding to this in different ways: while some teams held firm to their mild to moderate remit, others were trying to support children with more complex needs. This support was formally provided by more experienced therapists within teams, although several EMHPs described managing higher levels of risk than they had been trained for.

### The effectiveness of low-intensity CBT

According to survey data gathered by the Department for Education in 2017, only 18% of education settings nationally were offering CBT to pupils before the programme (Marshall et al., 2017). MHSTs were, therefore, increasing the range of mental health interventions children could access in their school or college. Despite this, there were concerns about the poor suitability of a standardised CBT approach for some groups (including children with learning difficulties, who were neuro-divergent, from certain ethnic minority communities and whose mental health problems were attributed to adverse family and/or socio-economic factors such as poverty or domestic abuse), and the lack of training of EMHPs to tailor support to individual and diverse needs:

*And in uni, thinking about interventions and things, they don’t teach you how to adapt things. Because I know their point of view is we shouldn’t really be working with [children with autistic spectrum disorder], but it happens. (Site 5, EMHP)*

Cultural and language barriers relating to the CBT approach were also described:

*I think, the intervention type being quite a Western offer frankly. CBT, you’ve got a cognitive ability to do it, you’ve got an articulation and a fundamental understanding of the English language and the English culture to be able to understand what mental health issues even are, what depression is, what anxiety is, and have a word to translate. It’s not translatable in our inner-city boroughs I don’t think, for every child and young person. (Site 3, MHST manager)*

Some expressed frustration at the perceived inflexibility of the service model:

*When I see a young person and I know I’ve got to stick within the realms of the model, that can be frustrating, when you know that you’ve got the skills to do something else with them, but you’ve got to stick to the model and the frame and not going outside of that. So, it’s very structured and it can be very fixed. (Site 4, EMHP).*

### Staff recruitment and retention

Recruitment to the initial national EMHP training programme had gone well and EMHPs generally enjoyed their training year. However, retaining EMHPs once in post was a major challenge, and reduced team capacity due to vacant EMHP posts was widely reported. Reasons given for this included the EMHP role being seen as a stepping-stone to other careers, lack of opportunities for career development and progression, mismatch between expectations and the reality of the role, high workloads and job demands:

*There are times when this role can become very draining, and very difficult to manage, particularly when you are overloaded with many complex cases and those sorts of things. (Site 4, EMHP)*

Senior staff were spending significant amounts of time on recruitment and induction activities, providing the more intensive supervisory support required for newly qualified EMPHs and helping to cover caseloads when there were vacancies. There was a widespread view that the programme must prioritise the issue of career development and progression opportunities for EMHPs, to reduce attrition and promote workforce stability.

In contrast to the generally positive experience of recruiting EMHPs, sites reported that appointing senior staff to MHSTs had been difficult throughout. One of the main implications of these recruitment problems was a lack of sufficient supervision for some EMHPs, another reason given for the loss of staff. Several factors were highlighted to explain the difficulties recruiting senior staff: national shortages in the mental health workforce, especially of more experienced professionals; salaries not being competitive in relation to other comparable roles; and the heavy emphasis on supervisory responsibilities, which was not attractive to those keen to continue practising therapeutically. MHSTs often recruited senior staff from local NHS services. While this had the potential to create knock-on staffing problems for those services, more positively it was facilitating knowledge sharing and relationship building between MHSTs and existing local providers.

## Discussion

Despite challenging circumstances, solid progress had been made in setting up MHSTs in the Trailblazer sites, with all 58 MHSTs operational (albeit in varying degrees) by January 2020, only 12 months after implementation started. There was, however, a lack of clarity at all levels regarding the scope and purpose of the programme and of MHSTs specifically, something which is crucial for successful implementation. This finding echoes previous studies, most notably the introduction of the Improving Access to Psychological Therapies (IAPT) service, on which key elements of the MHST programme are based (Burn et al., 2020; Parry et al., 2011). Relatedly, high staff turnover rates have been reported for low-intensity therapist roles in IAPT, driven by many of the same factors reported here for EMHPs, including workload pressures, inadequate supervision and lack of career progression opportunities (Steel et al., 2015; Westwood et al., 2017). This suggests there were missed opportunities to learn from the IAPT experience, in order that key implementation challenges could be anticipated and addressed at the programme design stage.

As has also been reported for IAPT and earlier initiatives involving primary care mental health workers, MHSTs were finding it difficult to practise within the remit of ‘mild to moderate’ mental health (Hutten et al., 2010; Rizq et al., 2010). The MHST model envisages that teams will help children and their families access other sources of support where MHSTs are unable to meet specific needs. But for this to happen there have to be services available to which they can refer, and this was not necessarily the case. Moreover, MHST staff were acutely aware of the growing demand for specialist mental health services, and of the long waiting times that children and their families faced trying to access them (Morris & Fisher, 2022). It was these children, with needs that were more serious than ‘mild to moderate’ but which failed to meet the threshold for specialist support, that education settings were generally most concerned about, and so it is perhaps unsurprising that MHSTs were being asked to work beyond their remit.

These findings provide further evidence of a remaining lack of services for children with moderate to severe mental health problems – the group McGorry (2022) has termed ‘the missing middle’ – and raise questions about what, if any, role MHSTs should have in addressing this gap. In the absence of a clear national steer, MHSTs were taking different decisions about whether to extend their scope, leading to variation in service provision across sites. There have been calls to expand the skill-mix and range of interventions offered by MHSTs, for example to include a qualified counsellor in every team, so that they can better meet the full range of mental health needs in schools and colleges that do not meet the threshold for specialist services (Barnardo’s, 2022). But without additional funding, MHSTs choosing to provide support to a wider range of children may find they can only do so by investing less time in their other functions, including those focused on mental health promotion and prevention.

This links to another study finding, which is that MHSTs have gravitated towards responding to mental ill-health, and away from universal whole-school activities. In the future, it may become harder still for MHSTs to prioritise such prevention-focused work in the context of the rising demand for mental health support (O’Shea, 2020). This has direct implications for the programme’s ability to address the ‘prevention gap’ in children’s mental health (Burstow et al., 2018), and to deliver the Green Paper’s ambition of “promoting positive mental health for all” (Department of Health & Department for Education, 2017). Ultimately, it may also limit the impact of the programme, given evidence that positive mental health cultures in education settings provide a supportive context in which targeted interventions are more likely to be effective (Cefai et al., 2021).

There was a widespread view that MHSTs must improve support for those groups whose mental health needs have been historically under-served, especially given that Covid-19 has further increased mental health inequalities among children. Highly structured, manualised interventions may be easier for therapists with less experience and more limited training to learn and deliver (Gee et al., 2021). However, early experience suggested that these standardised interventions were not working well for all children, findings consistent with wider evidence that adaptation is required to improve the suitability and effectiveness of CBT for some groups (Naeem, 2019; Spain & Happé, 2020; Sze & Wood, 2008). The need for flexibility in the service model and further training to equip EMHPs with skills and resources to adapt approaches and work in more culturally sensitive and trauma-informed ways was clearly apparent. All sites were providing additional ‘on the job’ training to EMHPs across a range of topics, with a strong focus on improving understanding of specific groups (e.g. neuro-divergent children) and how to tailor interventions to best meet their needs. Future research could usefully explore whether this training is helping to address EMHPs’ knowledge and skills gaps and, in so doing, is improving the suitability and effectiveness of MHST services for under-served groups.

## Study limitations

This study focused on the first 25 sites implementing MHSTs. These sites were chosen for characteristics thought likely to drive rapid progress and learning and therefore the findings from this evaluation may not be reflective of experiences across the programme as a whole. Efforts were made by the evaluation team to secure participation across different groups and at different levels of the programme, but undertaking fieldwork during Covid-19, including periods of national/local lockdown, was challenging. Some groups were less well represented in interview samples, including staff from education settings and specialist NHS mental health services.

## Conclusion

There is a strong rationale for investing in mental health prevention and support within education settings, and interviewees universally welcomed the creation of MHSTs. This study raises questions about MHSTs’ scope, what role they should play in addressing remaining gaps in mental health provision, and how EMHPs in particular can develop the skills to work effectively and meet the needs of diverse groups. Given the growing number of children experiencing mental health problems, answering these questions is more important now than ever.
